# Development and Theoretical Underpinnings of the PRIORITY Intervention: A Parenting Intervention to Prevent Disordered Eating in Children and Young People With Type 1 Diabetes

**DOI:** 10.3389/fcdhc.2022.822233

**Published:** 2022-02-17

**Authors:** Nicola R. O’Donnell, Rose-Marie Satherley, Mary John, Debbie Cooke, Lucy S. Hale, Rose Stewart, Christina J. Jones

**Affiliations:** ^1^ School of Psychology, University of Surrey, Guildford, United Kingdom; ^2^ Research and Development Department, Sussex Education Centre, Sussex Partnership NHS Foundation Trust, Brighton & Hove, United Kingdom; ^3^ School of Health Sciences, University of Surrey, Guildford, United Kingdom; ^4^ Wrexham Maelor Hospital, Betsi Cadwaladr University Health Board, Wrexham, United Kingdom

**Keywords:** Type 1 diabetes, disordered eating, children and young people (CYP), parents and caregivers, prevention, psychoeducation intervention, behaviour change interventions

## Abstract

Children and young people (CYP) with type 1 diabetes (T1D) are twice as likely to develop disordered eating (T1DE) and clinical eating disorders than those without. This has significant implications for physical and mental health, with some eating disorders associated with repeated diabetic ketoacidosis and higher HbA1c levels, both of which are life threatening. There is currently limited psychological support for CYP and families with T1D but increasingly, policy and practice are suggesting disordered eating in T1D may be effectively prevented through psychological intervention. We describe the development and theoretical underpinnings of a preventative psychological intervention for parents of CYP aged 11-14, with T1D. The intervention was informed by psychological theory, notably the Information Motivation Behaviour Skills model and Behaviour Change Technique Taxonomy. The intervention was co-developed with an expert advisory group of clinicians, and families with T1D. The manualised intervention includes two online group workshops, and supplementary online materials. The intervention continues to evolve, and feasibility findings will inform how best to align the intervention with routine care in NHS diabetes teams. Early detection and intervention are crucial in preventing T1DE, and it is hoped that the current intervention can contribute to improving the psychological and physical wellbeing of young people and families managing T1D.

## Background

Effective management of type one diabetes (T1D) places high treatment demands on the individual (1). Monitoring of carbohydrate intake with corresponding insulin dose adjustment and administration is an integral part of that treatment. Given this detailed focus on food, children and young people (CYP) are inherently more susceptible to developing unhelpful or negative relationships around food (2) CYP with T1D develop the cognitive capability to consider that insulin administration can impact weight (3). They are twice as likely to develop disordered eating (T1DE) and clinical eating disorders than people without T1D (4). Approximately 39% of CYP with T1D will develop disordered eating, and 7% will develop a clinically significant eating disorder, compared to only 3% of CYP without T1D (5). These eating behaviours (here on referred to as disordered eating) are costly to both the individual, family, and the health system, resulting in negative progression of T1D in terms of blood glucose fluctuations, repeated hospital admissions, impaired well-being, and early mortality (4, 6).

Prior to diagnosis of T1D, many individuals exhibit extreme weight loss that is quickly restored upon commencement of insulin therapy (7).This rapid weight gain, sometimes leading to significantly altered appearance, can result in lower self-esteem, body dissatisfaction and weight preoccupation (8). Furthermore, it can impact social interactions and relationships, with the potential for others commenting on their altered appearance and impacting body image perceptions (9). Combined with the fact that CYP are already managing the diagnosis of a life-long health condition and having to develop complex self-management and behavioural skills, it is not surprising that T1DE can develop. The focus on carbohydrate counting, whilst beneficial to attain euglycaemia (10) can lead to several psychological difficulties with food, impacting both individual and family dynamics. Foods containing carbohydrate can start to be perceived as ‘bad’ and thus avoided or prohibited within families. This can lead to heightened anxiety around mealtimes and increased tension between CYP and their parents (11), with CYP feeling pressured to avoid certain foods or eat ‘forbidden’ carbohydrates in secret. In addition, when CYP are experiencing hypoglycaemic episodes, it is recommended that they self-treat by eating rapid acting carbohydrate to restore their blood glucose levels (12). Although necessary, this intervention can add further complexities to CYP’s relationship with food. This could mean that a CYP overeats when they are hypoglycaemic or develop stringent rules around restriction that impacts the treatment of their low blood glucose levels. Additionally, peer relations can also impact T1D, with adolescence a time when eating out and drinking alcohol with friends becomes commonplace. This social context can add complications to a CYP’s relationship with diet, body image and T1D and must therefore be considered when designing an intervention for this population (13).

A recent Diabetes UK Position Statement identified several key gaps in the evidence base that might help improve mental well-being for CYP with T1D, one of which was supporting people with T1DE (6). Increasingly, specialist services are being developed for those with T1DE, and there are currently three specialist programmes in the United Kingdom to address this issue. Despite the availability of these specialist programmes, all focus on clinically significant eating disorders, as opposed to preventing disordered eating (14, 15). This is problematic as research suggests that preventative, early intervention is crucial in achieving better outcomes for this population, both in terms of eating disorders and the development of long-term serious T1D complications (16). Additionally, a recent systematic review of interventions addressing T1DE concluded that family-based interventions, including both diabetes management and eating disorder symptom content, were most effective in reducing eating disorder symptomology in T1D (9).

Guidelines for prevention of disordered eating in T1D have recommended interventions that focus on self-esteem, body acceptance and flexible approaches to food and meal planning, in line with diabetes management. Evidence from disordered eating in the general CYP population indicates that parental involvement is essential in the prevention of and recovery from disordered eating (17). This recommendation sees parents as a key resource, whereby improving parental confidence and knowledge increases effective management of their CYP’s eating difficulties as early as possible and supports them towards recovery (18). This approach also highlights early signs of disordered eating and provides families with deeper understanding as to how disordered eating negatively impacts a family as a unit and provided tools to prevent the maintenance of disordered eating within the family. There is widespread support that development of psychoeducation and behaviour change interventions should be informed by psychological theory and evidence of effectiveness (19). Despite this, psychological constructs and the development of psychological theory to explain the missing facets of interventions are often overlooked. They are therefore lacking from many interventions targeting physical health conditions. Theory can help researchers identify constructs that are related to a specific behaviour or psychological issue and therefore select appropriate targets for intervention (20). This paper provides a detailed overview of the development of a theoretically-informed complex intervention for preventing T1DE, adhering to the UK Medical Research Council (MRC) guidelines on developing and evaluating complex interventions (21). Through highlighting the specific behavioural components of the intervention, it is hoped that its effectiveness will be easily tested and replicable. The format used also aims to act as guidance for clinicians to utilise similar methods to develop solutions to other clinical challenges requiring intervention.

## PRIORITY Intervention Development

To address the aforementioned problems, this paper describes the development of a theoretically informed psychological intervention to prevent disordered eating in CYP with T1D. The PaRent InterventiOn to pRevent disordered eating in children with Type 1 diabetes (PRIORITY) is a complex psychoeducational intervention for parents of CYP with T1D. The manualised intervention consists of two, online, 120-minute psychoeducation workshops are supplemented with an interactive, online resource. Given the preventative nature of this intervention, this programme is aimed at any family with a CYP living with T1D, between 11-14 years, not just those expressing concern around disordered eating. The intervention uses accessible and engaging delivery methods informed by existing disordered eating prevention programmes and evidence-based psychological and behavioural literature (22–25). Specifically, the PRIORITY intervention drew on psychological theories of behaviour change, namely the Information-Motivation-Behaviour Skills (IMB) framework (26). Future work will assess whether this intervention improves parent and child outcomes, as well as the level of therapist skills to deliver the intervention in routine clinical settings (27).

### Psychological Theory: The Information-Motivation-Behaviour Skills Framework

A systematic process informing intervention development was utilised, advocated by Hawkins and colleagues’ guidelines on developing co-produced public health interventions (28). The process was underpinned by prior research, psychological theory, and extensive consultation with an expert advisory group. The IMB framework provided a useful model and has been used across a range of chronic conditions, including T1D (29). To engage in health behaviours, in this case to prevent T1DE, an individual must be informed about the risks of the behaviour, motivated to address these risks, and have the skills and confidence to address these risks across various situations. Interventions informed by the IMB model show promising effects within the context of T1D. For example, one RCT found that IMB model-based interventions targeting risk behaviours and obstacles to T1D management effectively improve glycaemic control for CYP with T1D (30). Another found that diabetes self-care apps utilising IMB theory were significantly more effective in increasing self-care social motivation (31). The current intervention targeted information, motivation, and behavioural skills, whilst building effective communication around T1D at the level of the child, family, and health provider. This aimed to lead to improvements in parental awareness of disordered eating and adolescent development, confidence to support their child’s transition to independent food choices, skills to promote healthy body image, and positive interactions around T1D management, and thus prevent the development of disordered eating.

Expanding upon this, the current intervention supplemented the IMB model with additional psychological principles known to influence disordered eating in T1D, namely the cognitive and emotional aspects of supporting a child with T1D. This included managing the loss of CYP independence (32), loss of parental confidence (33), and hypervigilance and fear in monitoring blood glucose (34). Other psychological and behavioural techniques were also integrated: peer support to increase parental motivation and acceptance, and the introduction of cognitive-behavioural techniques to increase ability to implement changes. This comprehensive approach allowed multi-level factors to be addressed to prevent the development of T1DE. Similar approaches have successfully been used in eating disorder prevention programmes in the general population (25).

### Co-Design With Experts

A group of multidisciplinary experts in T1D was established to ensure adherence to the co-design approach. Experts were purposively selected from a range of health service networks and patient public involvement groups, to ensure variability of background and experiences. The expert advisory group consisted of 12 members and included families of children with T1D (n=4), and healthcare professionals working with T1D families, namely paediatric diabetes specialist nurses (n=3), clinical psychologists (n=2), a diabetes specialist dietitian, a systemic therapist, and a consultant paediatrician. The research team took part in a series of iterative individual discussions with experts throughout the co-design process. Discussions were informed by three systematic reviews that identified the psychosocial determinants of disordered eating in T1D, the impact of interventions addressing T1DE, and the impact of parent interventions on preventing disordered eating in adolescents without diabetes (5, 35, 36). Discussions were also guided by the in-depth collection of qualitative work of Goebel-Fabbri, detailing the experiences of 25 women with type one diabetes, who identified themselves as recovered from an eating disorder (37).

**Figure 1:**
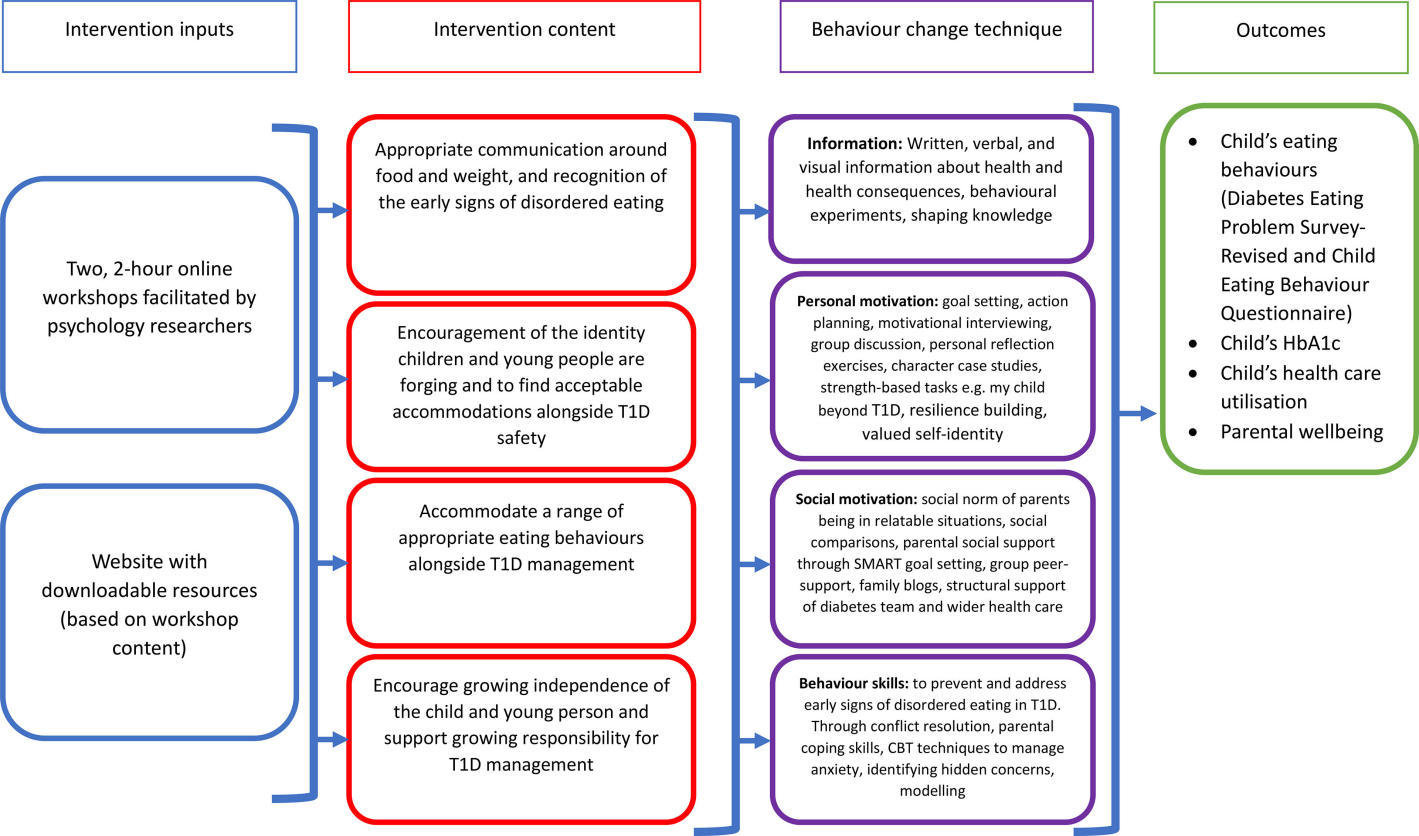
Logic model detailing intervention components and impact pathways. Intervention activities have been mapped onto the Information-Motivation-Behavioural Skills Model.

During initial conversations, experts described their knowledge and experience of disordered eating in T1D, reflected on common themes extracted from existing literature, identified existing successful and unsuccessful approaches to prevent and/or address T1DE, and highlighted gaps in clinical practice. Findings were broken down into specific behaviours, focusing on those that could be modified through parent interventions, selecting target behaviours to focus upon. Psychological determinants of these behaviours were then mapped onto domains of the IMB model to inform intervention content. A logic model of the intervention was developed which included areas of change that could be targeted by the programme; these were hypothesised to prevent the development of T1DE in CYP (**Figure 1**). This also included potential moderating factors, such as duration of T1D diagnosis, or parental ED, which may impact parental acquisition of IMB skills.

### Resulting Intervention Content

Intervention content was centred around four key parental behaviours that experts deemed important for preventing T1DE that are outlined below. Utilising the Behaviour Change Technique Taxonomy (24), these were mapped onto the IMB model to determine appropriate mechanisms for intervention delivery. This was further informed by the expert panel. Potential moderating factors hypothesised to affect parental acquisition of IMB skills were also considered when stratifying participants. These included duration of T1D diagnosis, insulin regimen, and parental ED (**Figure 1**).

#### Appropriate Communication Around Food and Weight and Recognising the Early Signs of T1DE

Experts reflected on the limited availability of specialist eating disorder services for CYP with T1D, with existing services focusing on the treatment of individuals misusing insulin to promote weight loss (14). Experts felt that although information around disordered eating and diabetes was essential to include, this information should not dominate intervention content. Clinicians reported seeing more subtle indicators of disordered eating, such as restriction, fears, and obsessions around exercising, and eating excessively in private. Therefore, experts felt that the focus of the intervention should address lower-level disordered eating, preventing CYP developing serious T1DE behaviours. It was important to the advisory group to include CYP without clinically significant eating disorders, as they felt that this is where interventions are often offered, overlooking individuals until they reach T1DE crisis point. This was reflected by families and clinicians who expressed concern about the limited availability of psychological support in diabetes services. To incorporate this with relevant literature (38, 39) the intervention content focuses on developing parental skills to promote a healthy body image, identify disordered eating, and manage risk. Families spoke about the challenges of discussing food and weight in the diabetes clinic, unsure of whether concerns beyond carbohydrate counting warranted support. It was apparent that many parents and CYP felt unclear about the role of the diabetes team and wanted the opportunity to talk about more than just food intake and glucose monitoring. This was equally important to the dietitians in the advisory group, who also wanted more holistic interaction with families, including discussion around the social role of food and how to manage this with T1D. An example of this was a parent sharing their anxiety about their child being invited to social events and needing to attend so they could monitor food intake and insulin requirements. As discussed, Advisory group discussions highlighted the need for psychological support in T1D teams to be holistic and ongoing. For this reason, the PRIORITY intervention includes information about the role of T1D clinicians and advice on how to start difficult conversations about disordered eating or any element of T1D. The intervention acts as a starting point to empower parents to initiate conversations with CYP about T1D, provide tools and strategies to promote CYP ownership of their condition and parental confidence in this, and work with clinicians for their child to achieve optimum physical and psychological wellbeing.

#### Encouraging the Identity CYP Are Forging and to Find Acceptable Accommodations Alongside Diabetes Safety

Families readily spoke about the precursors to disordered eating including identity concerns and family conflict. For example, parents spoke of their CYP increasingly wanting to go to sleepovers, school trips, or other events on their own. Health providers highlighted the importance of a strong identity as a protective factor in T1DE, suggesting that CYP needed to accommodate T1D within their sense of self, alongside aspects of their identity outside of T1D. This ability to consider individual and family identity, and where T1D fits in with this, plays a well-established role in treatments for disordered eating, and self-management of T1D.

The intervention developed utilised a narrative approach (40) to expand on lived experiences, developing two fictitious families who illustrate various T1D scenarios throughout the intervention. It was hoped that through developing situations for our characters to navigate, that this would externalise T1D and allow families to see it as a part of, rather than their whole identity. This is a technique commonly used in narrative therapy as it allows families to be creative and generate distance between themselves and health conditions. Shown to be feasible as an approach for adolescents with T1D (41), it is also hoped that this approach would empower and unite families, enabling discussion about their feelings more easily. The purpose of these characters is to highlight the thread of communication throughout the workshops and website, as this is the key skill that underpins all the aims, activities, and discussions in this intervention. It is also hoped that these scenarios would increase parental motivation and skills, creating a social norm around parenting CYP with T1D and encouraging resilience building and action planning (42).

Families felt it was important to develop an intervention that is accessible for any family with a CYP with T1D, not just those with pre-existing eating disorder concerns. For this reason, the intervention was developed to provide tools to manage general anxiety, navigate typical adolescent concerns, such as identity, and focus on problem-solving techniques (43–45). For sustainability across services, the intervention needed to be low intensity and manualised for fidelity. This would not only improve accessibility but ensure integration of low intensity psychological support into diabetes multi-disciplinary teams. This, in turn, would increase levels of awareness of T1DE in the general diabetes population as well as equip parents with information on how to identify DE and access support.

#### Accommodate a Range of Appropriate Eating Behaviours Alongside Diabetes Management

Acknowledgement of the challenge of balancing T1D management alongside healthy eating behaviours was crucial. Although not a dietary intervention, it was important to discuss parental concerns around diet and the impact that this has on psychological wellbeing. Placing parents in the position of experts, they are reminded that they and their CYP are highly skilled in managing the complexities of diabetes self-management and integrating this into their daily lives. Through discussing the different roles of food (e.g. practical, social, emotional, cognitive) and considering parents’ own relationship with food, the intervention supports parents to better identify any disordered eating in their family and CYP with T1D, both now and in the future. It was important to families that there was a focus on how to be flexible around food, citing that when diagnosed with T1D there is a risk of becoming rigid and controlling around dietary intake. Working alongside families to develop and enhance a range of skills is crucial, modelling flexibility and supporting parents to set boundaries aims to encourage the accommodation of a range of appropriate eating behaviours that suit a whole family alongside T1D (40, 41, 46–48).

#### Encouraging Growing Independence of the Child or Young Person, and Growing Responsibility for Diabetes Management

Developing parental (and anticipated subsequent CYP) confidence through the provision of new skills was key to achieving the intervention outcomes. The PRIORITY intervention website and workshops emphasise that the tools that they need may change as their CYP grows and forges their independence, depending on their experiences and circumstances. The intervention provides information to parents to highlight how common it is for parents and their CYP to disagree about independence, both about T1D and ‘typical’ adolescent issues. Facilitator and peer active listening and support will allow parents to hear differing opinions about independence and how much to give a child at different ages and stages. This will be supported by the inclusion of parents whose CYP have been diagnosed for varying amounts of time, with more experienced parents able to offer their experience to support to those more newly diagnosed (49). It is also important for parents to feel reassured that it is natural to want to keep their child safe, and that overprotection usually comes from a place of care (50). Managing expectations is also addressed, with parents given the opportunity to discuss their current reality as well as reflect on their hopes for the future. Providing parents with the information and tools to look after their own mental health is also key, and providing psychoeducation around feelings such as anxiety, as well as the potential impact on thoughts, feelings, and behaviour, will increase self-awareness and understanding as to why certain situations may feel challenging. The skills and techniques that the intervention uses to prevent T1DE drew on cognitive behavioural techniques such as cognitive restructuring and problem solving. Conflict resolution and managing family identity alongside T1D, identified through systemic literature and the co-design approach were also included (5, 51, 52). These are embedded alongside character scenarios which ask parents to consider key questions to reflect on the character/their own experiences.

## Next Steps

A feasibility randomised controlled trial of the PRIORITY intervention is being completed with 70 parents recruited from NHS settings and third sector organisation to determine acceptability and feasibility of the approach (27). Alongside analyses of outcomes, qualitative interviews will be conducted with participants to ensure a definitive trial is acceptable and reflective of the needs of the population of parents of CYP with T1D. The co-development of the project has allowed us to establish several recommendations for the development of preventative interventions for T1DE.

## Summary and Implications for Practice

Early detection and intervention are crucial in preventing T1DE. Psychoeducational programmes can be an asset to diabetes clinical care as they require minimal effort and staffing to deliver and introduce topics that can be explored in more detail during clinical practice (53–55). The PRIORITY intervention has been specifically designed to be delivered as a low-intensity, manualised psychological intervention. It is hoped that this flexibility will support ease of delivery, keep costs low, and eventually develop the skills of all multi-disciplinary team members pending further evaluation of delivery by psychologists. If the intervention is deemed helpful when delivered by clinicians with training in psychological theory and approaches, it is hoped that delivery will be optimised though upskilling and training other members of diabetes care teams. The PRIORITY intervention is preventative and applicable to families without specific disordered eating concerns, which is hoped to reduce burden on specific eating disorder services if T1DE can be managed within secondary care diabetes teams. Programmes such as the DAFNE model (56), which is implemented in routine adult diabetes care, demonstrate the success of theoretically-informed psychoeducation programmes in this population. If shown to be feasible the current preventative intervention could be similarly embedded into routine practice within diabetes services. Through the provision of this low-intensity cognitive behavioural and narrative, theoretically informed family intervention, we hope to provide much-needed support for the psychological and physical wellbeing of CYPs and families with T1D.

## Data Availability Statement

The original contributions presented in the study are included in the article/supplementary material. Further inquiries can be directed to the corresponding author.

## Author Contributions

CJ, MJ, DC, LH, and RS designed the study. NO’D and R-MS were active in the co-design with experts with NO’D. NO'D, RM-S and CJ guided the development of the intervention via the theoretical methodology. NO’D, wrote the first draft of the manuscript. R-MS and CJ critically advised on important intellectual content and contributed to revising of the manuscript, with input from MJ, DC and LH. All authors contributed to the article and approved the submitted version.

## Funding

This work was supported by Diabetes UK (award number 19/0006123).

## Conflict of Interest

The authors declare that the research was conducted in the absence of any commercial or financial relationships that could be construed as a potential conflict of interest.

## Publisher’s Note

All claims expressed in this article are solely those of the authors and do not necessarily represent those of their affiliated organizations, or those of the publisher, the editors and the reviewers. Any product that may be evaluated in this article, or claim that may be made by its manufacturer, is not guaranteed or endorsed by the publisher.
